# Perioperative, functional, and oncological outcomes after cryoablation or partial nephrectomy for small renal masses in solitary kidneys: a systematic review and meta-analysis

**DOI:** 10.1186/s12894-024-01406-x

**Published:** 2024-01-24

**Authors:** Ying Liu, Li Wang, Er-hao Bao, Lei Wang, Jia-hao Wang, Lin Yang, Ping-yu Zhu

**Affiliations:** Department of Urology, Affiliated Hospital of North Sichuan Medical College, Nanchong, Sichuan China

**Keywords:** Partial nephrectomy, Cryoablation, Small renal masses, Solitary kidney, Meta-analysis

## Abstract

**Aim:**

This study aims to compare the perioperative, functional, and oncological outcomes of cryoablation (CA) and partial nephrectomy (PN) for managing small renal masses in patients with solitary kidneys. The study seeks to assess the efficacy and safety of both interventions, evaluating their impact on kidney function and their ability to mitigate cancer recurrence.

**Methods:**

Searches were systematically conducted on PubMed, Scopus, EMBASE, SinoMed, and Google Scholar, identifying seven observational studies. Statistical analysis was performed using Stata v.12.0 and Review Manager version 5.2. Results for dichotomous variables are expressed using odds ratios, and weighted mean differences are used for continuous variables.

**Results:**

Our findings revealed that patients undergoing CA experienced significantly shorter operative time (*p* < 0.0001), reduced estimated blood loss (*p* < 0.00001), a shorter length of stay (*p* = 0.0001), and fewer postoperative complications (*p* = 0.02) compared to those undergoing PN. Although the CA group exhibited a lower transfusion rate (*p* = 0.69) compared with the PN group, the difference was not statistically significant. The combined data analysis demonstrated a significantly lower increase in serum creatinine levels after surgery in the CA group compared with the PN group (*p* = 0.003). Similarly, there was a noteworthy decrease in the estimated glomerular filtration rate after surgery in the PN group compared with the CA group (*p* < 0.0001). While not statistically significant, the CA group showed a lower postoperative dialysis rate (*p* = 0.11). Regarding oncological outcomes, the analysis revealed no significant differences between CA and PN concerning local recurrence (*p* = 0.2) and distant metastasis (*p* = 0.12), respectively.

**Conclusions:**

Our analysis indicates comparable efficacy between PN and CA in controlling tumour recurrence and metastasis. However, CA is associated with superior preservation of renal function, significantly enhanced perioperative outcomes, and fewer postoperative complications. Based on our data, it can be inferred that the scope for applying CA might be expanded to encompass more patients seeking a less invasive treatment option.

**Supplementary Information:**

The online version contains supplementary material available at 10.1186/s12894-024-01406-x.

## Introduction

The term " small renal mass” encompasses a diverse group of tumours, ranging from benign and asymptomatic growths to malignant lesions with metastatic potential. Renal cell carcinoma constitutes 2–3% of all cancers, with its incidence steadily increasing each year [1, 2] Guidelines recommend PN as the preferred management option for small renal masses [3].

However, the management of patients with a solitary kidney places a significant emphasis on renal function. As the sole functioning kidney in the body, any compromise in its function can have serious consequences on overall health [4]. .The ability of the kidney to filter waste products and maintain fluid and electrolyte balance is essential for normal bodily functions. In individuals with a solitary kidney, a decline in renal function increases the risk of developing chronic kidney disease (CKD) or end-stage renal disease [5]. Therefore, monitoring and preserving renal function is of paramount importance in treating patients with a solitary kidney, aiming to prevent further complications and ensure optimal outcomes.

Therefore, it is noteworthy that the introduction of cryoablation (CA) techniques has ushered in a new era in the management of small renal masses in a solitary kidney [6, 7]. The objective of treating small renal masses in a solitary kidney, whether through partial nephrectomy (PN) or CA, extends beyond tumour elimination to minimise perioperative complications, preserve renal function, and lower postoperative recurrence rates [8]. Consequently, identifying the most appropriate treatment strategy for small renal masses necessitates the consideration of various factors such as perioperative concerns, tumour outcomes, and renal function. However, the existing literature exploring the relationship between PN and CA is limited. To date, only one systematic review has investigated the effectiveness of CA and PN in treating small renal masses in a solitary kidney [9]. However, this review encompassed a limited number of studies and participants. Therefore, it is imperative to undertake a comparative study between PN and CA to establish the most effective treatment approach for small renal masses in a solitary kidney.

## Methods

### Protocol and guidance

This study followed the guidelines outlined in Preferred Reporting Items for Systematic Reviews and Meta-Analyses [10] (refer to Table S1) and was pre-registered in the International Prospective Register of Systematic Reviews database (CRD42023426806). Complying with these standards not only ensures transparency and precision in reporting but also aids in mitigating bias and enhancing the reproducibility of the study findings.

### Search strategy

This study encompassed literature available on PubMed, Scopus, EMBASE, SinoMed, and Google Scholar until 22 March 2023. The search used Medical Subject Heading terms and keywords, including “Ablation” OR “Cryoablation”, “Partial Nephrectomy”, and “Solitary Kidney”. Meanwhile, there were no restrictions on publication year or language.

### Inclusion and exclusion criteria

The eligibility criteria were established based on the Population, Intervention, Comparison, Outcomes, and Study framework. Specifically, P refers to patients with small renal masses in a solitary kidney; I involves those undergoing PN; C compares PN to CA; O includes one or more of the following outcomes: perioperative, renal functional, and oncological outcomes; S encompasses both prospective and retrospective cohort studies, case-control studies, and randomised controlled trials (RCTs). Non-comparative studies, editorial comments, unpublished studies or comments, and studies lacking data were excluded. This systematic approach ensures that only relevant studies meeting specific criteria are included in the analysis, ultimately leading to more accurate and meaningful results.

### Data extraction and items

Two independent reviewers (LY and EH) extracted data using an Excel spreadsheet. The collected information encompassed: <1 > basic information, including study design, number of patients, sex distribution, age range, body mass index (BMI), preoperative serum creatinine (sCr) levels, preoperative estimated glomerular filtration rate (eGFR), preoperative CKD rate, length of follow-up, and tumour size; <2 > surgical outcomes, including operative time (OT), estimated blood loss (EBL), length of stay (LOS), blood transfusion, and postoperative complications; <3 > renal functional outcomes, comprising the increase in sCr after surgery, the decrease in the eGFR after surgery, and postoperative dialysis rate; <4 > oncology-related outcomes, specifically the local recurrence rate and distant recurrence rate. This systematic approach to data extraction ensures the comprehensive and consistent capture of all relevant information. In instances of disagreement, consultation with another researcher (WL) was employed to reach a consensus.

### Risk of bias and certainty in evidence

Two independent reviewers evaluated the risk of bias in each study using the Risk Of Bias In Non-randomised Studies - of Interventions (ROBINS-I) tool for non-randomised trials. The quality of evidence was appraised using the Newcastle–Ottawa scale (NOS). In case of discrepancies, a consensus was achieved through discussion and mutual agreement.

### Statistical analysis

Statistical analysis was performed using Stata v.12.0 (TX, USA) and Review Manager version 5.2 (Oxford, UK). Medians and quartiles were converted to means and standard deviations using tables provided by Luo et al. [11] ( and McGrath et al. [12]. Odds ratios (ORs) were used to express the results for dichotomous variables, while weighted mean differences (WMDs) were employed for continuous variables. Our findings were reported with 95% confidence intervals (CIs) for all outcomes.

### Publication bias

Typically, publication bias analysis was omitted when the number of included studies was below 10 due to insufficient statistical power [13, 14].

## Results

### Baseline characteristics

**Fig. 1:**
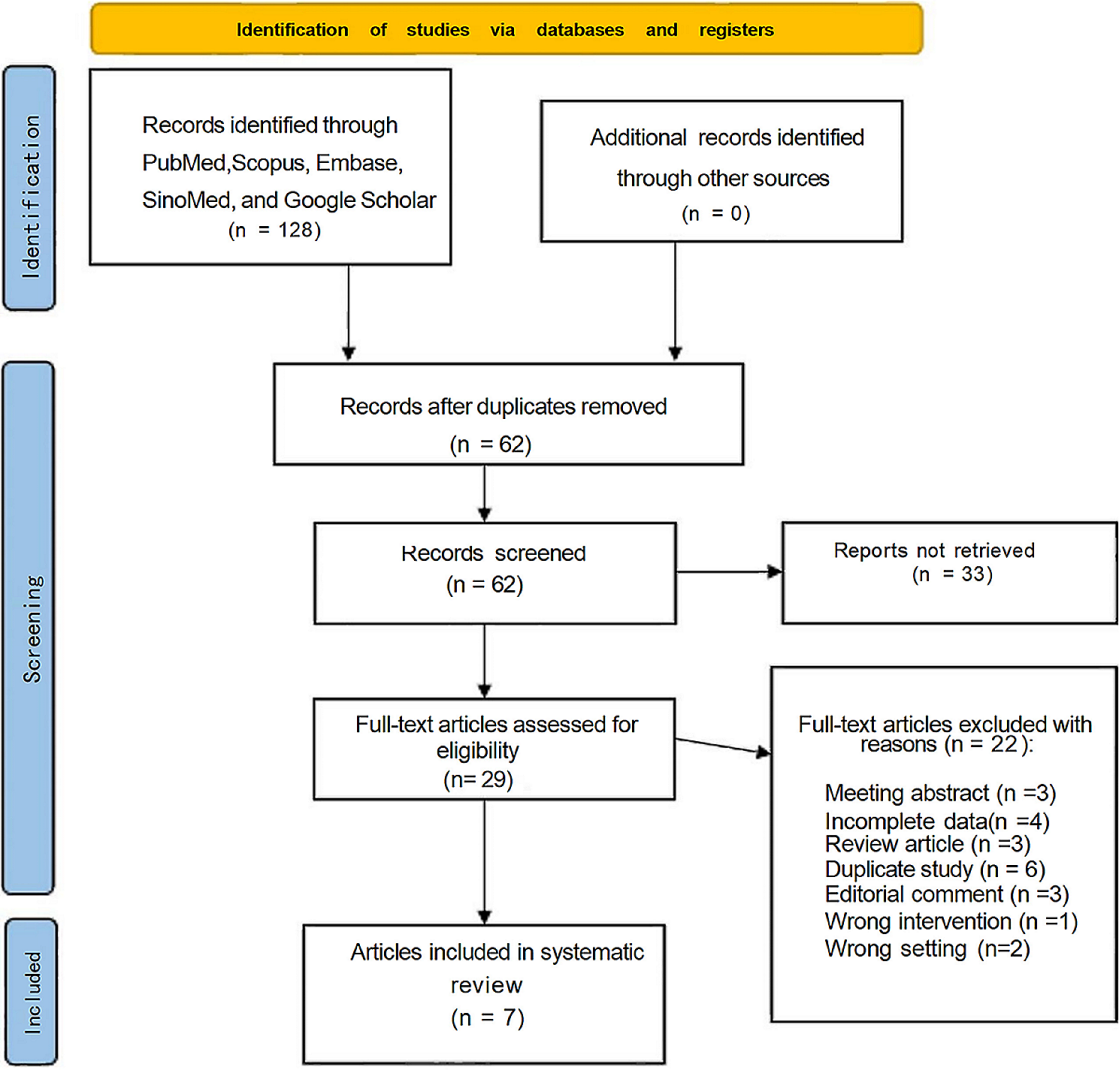
The Preferred reporting items for systematic reviews and meta-analyses flowchart

Following the initial selection of the search strategy, 90 publications were identified as relevant to our study. After eliminating duplicates and conducting a comprehensive review of titles, abstracts, and full texts, seven controlled studies were ultimately included. Figure 1 illustrates the PRISMA flowchart outlining this process. The selected studies spanned from 2002 to 2022, with two being prospective [15, 16] and five being retrospective [17–21]. All studies were performed in the United States of America. Among the patients, 1069 patients (76.4%) underwent PN, while 331 patients (23.6%) underwent CA, respectively. Table 1 presents a summary of the basic study information. Notably, there were no statistically significant differences in BMI (*p* = 0.71), proportion of males (*p* = 0.27), prevalence of preoperative CKD (*p* = 0.06), or length of follow-up (*p* = 0.6). However, significant differences were observed in preoperative sCr (*p* = 0.05), preoperative eGFR (*p* = 0.0002), age (*p* = 0.0009), and tumour size (*p* = 0.001). These summarised results are presented in Table 2. Additionally, Table S2 provides details on tumour histological subtype, stage, and Furman grade.

**Table 1:** Basic information of the included studies

Study	Design	No. of	Preoperative renal function			Age (year)	Male (%)	BMI (kg/m^2^)		Tumor Size (cm)	Follow-up (month)	Quality Score
		cases, type	Creatinine	eGFR	CKD (%)							
Turna2009	P	36 PN	1.2	65	63.9	60.3	58	30.5	3.7		42.8	8
		36 CA	1.4	52.3	55.6	64.1	64	31.3	2.5		28.0	
Goyal2011	R	15 PN	1.5	55.1	60	65.2	86.7	NA	3.4		30.8	9
		23 CA	1.3	54.6	65.2	68.4	56.5	NA	2.5		31.2	
Haber2011	R	48 PN	1.2	61.6	NA	60.6	25	30.1	3.2		42.7	8
		30 CA	1.5	53.8	NA	60.9	22	31.5	2.6		60.2	
Kamol2013	R	33 PN	1.2	62	42	60	67	29.0	3.0		NA	8
		43 CA	1.3	57	70	64.4	81	29.0	2.3		NA	
Bhindi2017	P	64PN	NA	60.2	NA	62.6	73	NA	3.6		NA	9
		54CA	NA	57.8	NA	64.6	75	NA	4.1		NA	
BeKSaC2022	R	31PN	NA	58	52	60.4	55	31	2.8		NA	8
		43CA	NA	56	70	67.3	72	28	2.1		NA	
Yasuda2022	R	842PN	NA	57.4	NA	64.7	64	29	4.0		58.7	9
		102CA	NA	52.6	NA	65.9	68	31	2.3		52.0	

P = prospective; R = retrospective; eGFR = estimated glomerular filtration rate; CKD = chronic kidney disease; BMI = Body mass index; CA = Cryoablation; PN = Partial nephrectomy; NA = not available

**Table 2:** Baseline comparison of patients

variable	No. of studies with available data	WMD/OR	95% CI	*P* value
male proportion (%)	7	0.85	(0.63,1.14)	0.27
BMI (kg/m^2^)	5	-0.33	(-2.11,1.44)	0.71
preop serum creatinine(mg/)	4	-0.14	(-0.29,0.00)	0.05
preop eGFR (ml/min/1.73 m^2^)	7	4.91	(2.34,7.47)	0.0002
preop CKD (%)	4	0.62	(0.38,1.03)	0.06
Follow -up time (month)	4	-5.72	(-27.07,15.63)	0.6
age (year)	7	-2.7	(-4.29, -1.10)	0.0009
tumour size (cm)	7	0.83	(0.32,1.33)	0.001

WMD = weighted mean difference; OR = odds ratio; CI = confidence interval; BMI = body mass index; eGFR = estimated glomerular filtration rate; CKD = chronic kidney disease;

### Perioperative effectiveness

**Fig. 2:**
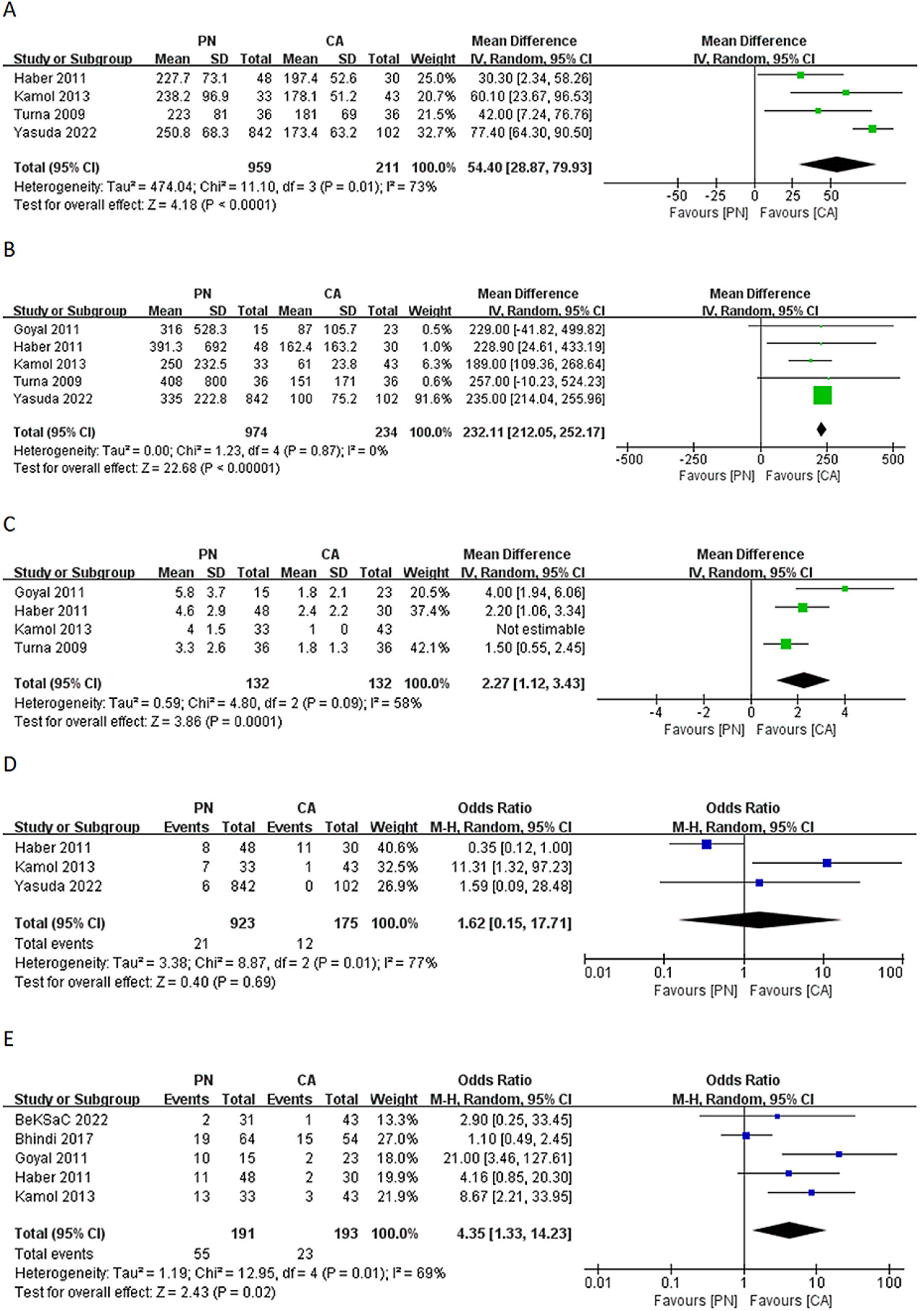
Comparison of partial nephrectomy and cryoablation: (**A**) operative time; (**B**) estimated blood loss; (**C**) length of stay; (**D**) transfusion rates; (**E**) postoperative complications

Four studies, encompassing 1170 patients (959 undergoing PN and 211 undergoing CA), reported on the OT. The pooled results demonstrated that CA significantly reduced OT compared to PN, with a WMD of 54.40 min (95% CI: 28.87 to 79.93 min; *p* < 0.0001) (Fig. 2A). In the case of EBL, five studies involving 1208 patients (974 undergoing PN and 234 undergoing CA) were assessed. The pooled results indicated that CA was associated with significantly lower EBL compared to PN, with a WMD of 232.11 mL (95% CI: 212.05 to 252.17 mL; *p* < 0.00001) (Fig. 2B). Examining the LOS, four studies (references 15–18) involving 264 patients (132 undergoing PN and 132 undergoing CA) were included. The pooled results revealed that CA was associated with a significantly shorter LOS compared to PN, with WMD of 2.27 days (95% CI: 1.12 to 3.43 days; *p* = 0.0001) (Fig. 2C). Regarding transfusion rates, three studies covering 923 patients undergoing PN and 175 patients undergoing CA were evaluated. The comparison indicated no significant difference in transfusion rates between PN and CA (OR: 1.62; 95% CI: 0.15 to 17.71; *p* = 0.69) (Fig. 2D). Postoperative complications were reported in 384 patients (191 undergoing PN and 193 undergoing CA) across five studies. The analysis demonstrated that the incidence of postoperative complications was significantly lower in the CA group compared with the PN group (OR: 4.35; 95% CI: 1.33 to 14.23; *p* = 0.02) (Fig. 2E).

### Renal functional outcomes

**Fig. 3:**
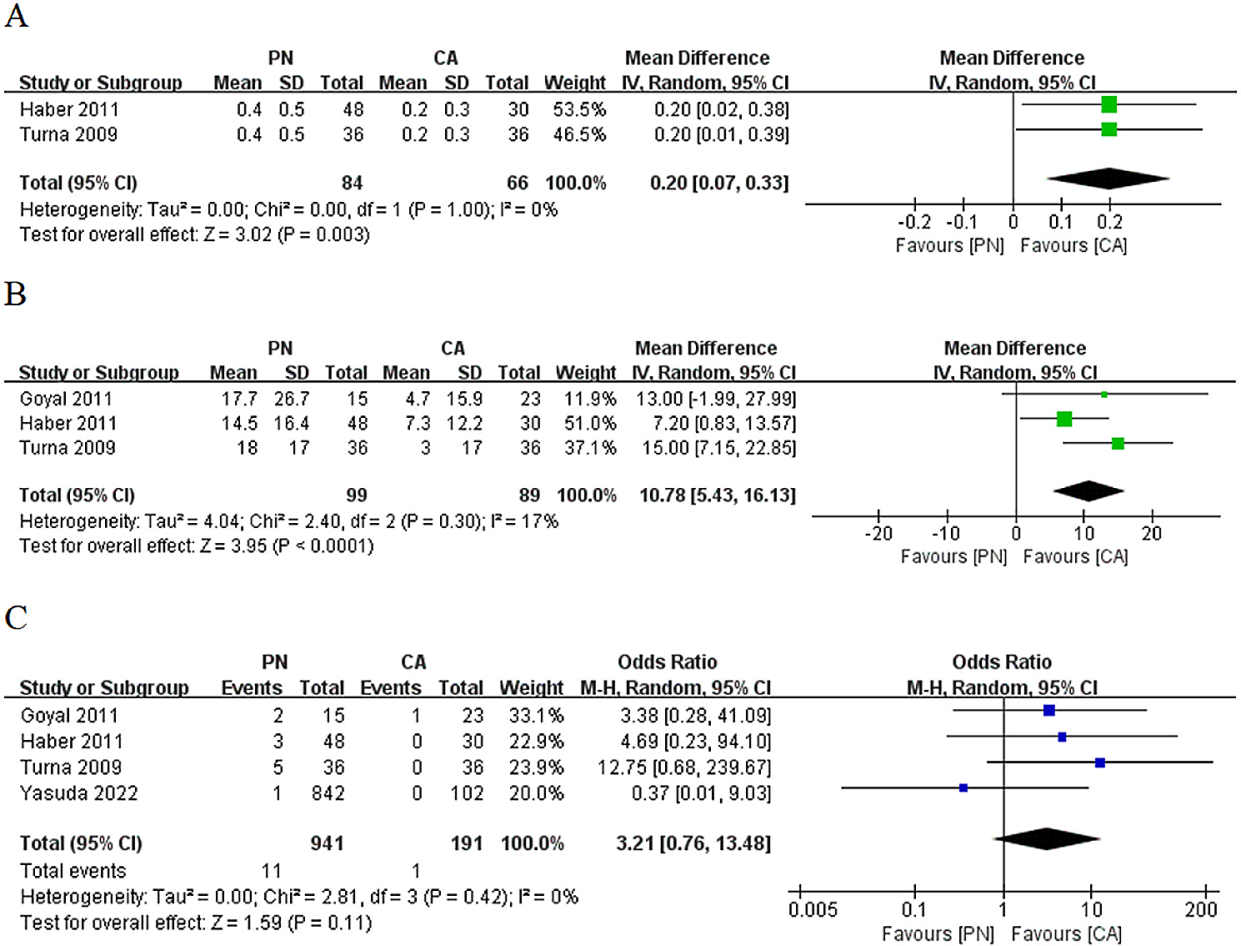
Comparison of partial nephrectomy and cryoablation: (**A**) increased serum creatinine after surgery; (**B**) decreased estimated glomerular filtration rate after surgery; (**C**) dialysis rate

The analysis of the included studies revealed that the increase in sCr levels after surgery was significantly lower in the CA group compared with the PN group (WMD: 0.20; 95% CI: 0.07 to 0.33; *p* = 0.003) (Fig. 3A). Additionally, the decrease in eGFR after surgery was significantly lower in the CA group compared with the PN group (WMD: -10.78; 95% CI: -5.43 to -16.13; *p* < 0.0001) (Fig. 3B). Furthermore, though not statistically significant, the postoperative dialysis rate was higher in the PN group than in the CA group (OR: 3.21; 95% CI: 0.76 to 13.48; *p* = 0.11) (Fig. 3C).

### Oncological outcomes

**Fig. 4:**
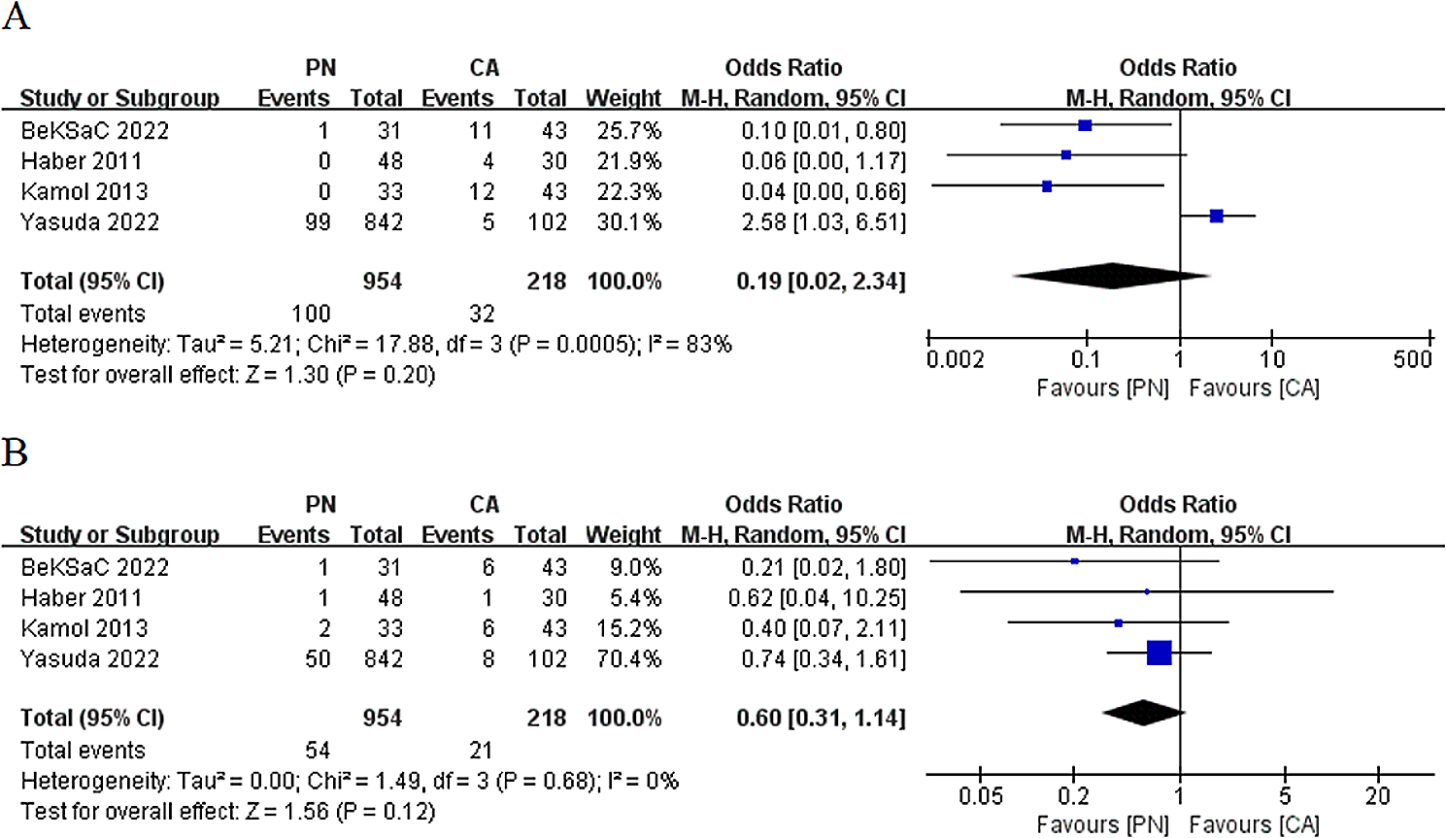
Comparison of partial nephrectomy and cryoablation: (**A**) local recurrence rate; (**B**) distant metastasis rate

The mean follow-up period for oncological outcomes after PN ranged from 28.7 to 58.6 months, whereas for CA, it ranged from 28 to 75.3 months. Four studies (references 17, 18, 20, and 21) provided data on the local recurrence rate and distant metastasis rate. The analysis indicated no significant difference between PN and CA concerning the local recurrence rate (OR: 0.19; 95% CI: 0.02 to 2.34; *p* = 0.2) (Fig. 4A) or distant metastasis rate (OR: 0.60; 95% CI: 0.31 to 1.14; *p* = 0.12) (Fig. 4B).

### Risk of bias and assessment of quality

The study period covered the years 2009 to 2022, and the application of the ROBINS-I tool indicated moderate overall bias in the study (as presented in Table S3). Furthermore, our examination demonstrated that all included studies were of moderate or higher quality, as evidenced by their NOS scores (> 5). Detailed evidence regarding the quality assessment is presented in Table S4.

### Analysis of sensitivity

While most studies exhibited low to moderate levels of heterogeneity, certain outcomes such as OT, transfusion rates, postoperative complications, and local recurrence rate presented high levels of heterogeneity (I^2^ > 60%). Sensitivity testing was conducted on a subset of studies demonstrating substantial heterogeneity to ensure the validity of these outcomes. It is important to note that this type of testing was not performed when comparing three or fewer studies. Upon individually excluding each included study and recomputing the overall mean difference, it was observed that excluding Yasuda’s [21] study resulted in a significantly lower local recurrence rate for CA compared to PN (OR: 0.07; *p* = 0.0003). This observation might be attributed to larger tumour sizes in the PN group in this particular study. Furthermore, the technical demands for doctors performing PN were higher. Moreover, the follow-up time in the PN group exceeded that in the CA group. The findings of the remaining studies remained relatively consistent.

## Discussion

Patients tend to opt for PN when they have better renal function, while those with poor renal function often lean towards CA [19, 22]. Additionally, older individuals and those with smaller masses are more inclined towards CA, whereas younger individuals and those with larger masses tend to choose PN [23]. This trend might be attributed to the less invasive nature of CA, which is associated with reduced damage to kidney function and is generally more tolerable for older patients with poorer kidney function. However, CA might face challenges in completely clearing large-diameter tumours. These observations align with the statistical findings. Compared with radical nephrectomy, PN allows the preservation of a portion of nephrons and renal function. However, the need to block the renal artery intraoperatively in PN might have unfavourable implications for renal function protection [24]. With advancements in CA technology, it has emerged as a preferred treatment option due to its ease of operation and minimal trauma compared to alternative methods [25].

### Perioperative outcomes and postoperative complications

Compared with PN, patients undergoing CA exhibited significantly lower OT, EBL and LOS. While the transfusion rate and postoperative complications were higher in the PN group compared with the CA group, the difference was not statistically significant. This can be attributed, on the one hand, to the greater complexity and difficulty associated with PN procedures [26]. On the other hand, patients with larger tumour diameters tended to opt for PN [27]. Despite the rapid development in the treatment of small renal masses through PN, the incidence rates of blood transfusion and postoperative complications remained higher than those for CA, although these differences were not statistically significant. Surgeons are more likely to choose CA over PN for patients with poor physical conditions, advanced age, compromised renal function, and small tumour diameters before surgery. Therefore, the adoption of minimally invasive and less invasive treatment approaches is expected to yield better perioperative outcomes [28].

### Renal functional outcomes

In cases involving patients with a solitary kidney, preserving renal function is paramount, as it stands out as one of the most significant risk factors for chronic kidney disease following renal surgery [29]. Diminished renal function independently predicts heightened hospitalisation rates, an increased incidence of cardiovascular events, and elevated mortality rates [30]. This article highlights the benefits of using CA to protect renal function. A notable advantage of CA over PN lies in its avoidance of blocking renal arteries, consequently reducing renal thermal ischaemia time and mitigating ischaemia-reperfusion injury. This, in turn, contributes to the preservation of renal function [31]. CA possesses the capability to selectively eliminate tumour tissue while preserving a greater portion of normal kidney tissue, thereby aiding in the maintenance of kidney function. CA is currently used for small renal tumours and holds promise for future applications. Conversely, PN poses technical challenges, requiring intracorporeal suturing under ischaemia, which could result in prolonged warm ischaemia times and decreased postoperative GFR [32].

### Oncological outcomes

Our analysis indicates that PN and CA exhibit comparable efficacy in controlling tumour recurrence and metastasis. However, a single study suggests that PN might be more effective than CA in terms of the local recurrence and distant metastasis rates [33]. This discrepancy could be attributed to instances where the mass morphology is irregular, and CA might not ensure complete coverage of the mass. Although PN can more thoroughly eliminate the tumour, it might also result in the removal of more normal kidney tissue. Despite the higher local recurrence and distant metastasis rates associated with CA compared with PN, the convenience and less traumatic nature of multiple CA procedures, along with the potential for preserving renal function and prolonging life, should be considered. Literature reports indicate that the overall survival rates of CA and PN in treating small renal masses in a solitary kidney are similar [34].

## Limitations

This study has several limitations. First, none of the studies included in the analysis were RCTs. The analysis predominantly relied on observational studies, rendering it susceptible to bias and confounding factors. Second, since the original studies did not classify the type of surgery, distinctions between various surgical approaches (laparoscopic vs. percutaneous vs. robotic) were not made. Third, analysis of the baseline data included in the study showed that patients in the CA group tended to be older, have smaller tumours, and have worse preoperative renal function, which may overestimate the advantages of CA in terms of complications, perioperative outcomes., In addition, the absence of long-term and follow-up control studies with large sample sizes prevented a comprehensive evaluation of the long-term prognosis of tumours. Therefore, it is imperative to conduct more extensive and high-quality RCTs to provide a more robust validation for the pooled results.

## Conclusions

PN proves to be an effective approach for managing small renal masses in a solitary kidney. On the other hand, CA emerges as a more minimally invasive and less invasive treatment for patients with compromised preoperative health, advanced age, and poor renal function, which is a preferable choice. In summary, the selection of any treatment modality should be guided by a comprehensive consideration of its advantages and disadvantages according to the specific circumstances of the patients, ensuring the choice of the most suitable treatment.

### Electronic supplementary material

Below is the link to the electronic supplementary material.

Supplementary Material 1

## Data Availability

The article and accompanying supplementary materials encompass all available data.
